# Determinants of COVID-19-related knowledge and disrupted habits during epidemic waves among women of childbearing age in urban and rural areas of the Malagasy Middle East

**DOI:** 10.1186/s12889-023-16931-x

**Published:** 2023-10-12

**Authors:** Sitraka Rakotosamimanana, Reziky Tiandraza Mangahasimbola, Rila Ratovoson, Rindra Vatosoa Randremanana

**Affiliations:** Institut Pasteur de Madagascar, Antananarivo 101, Madagascar

**Keywords:** Madagascar, COVID-19, Maternal and child health, Knowledge

## Abstract

**Background:**

With regard to the coronavirus disease (COVID-19) pandemic in Madagascar, little is known about the knowledge, the perceptions and the impacts of this disease on women of childbearing age. People’s knowledge of COVID-19 can have an impact on their attitudes towards seeking care. The aim of the current study is to determine the knowledge of COVID-19 and associated determinants among women of childbearing age in Moramanga.

**Methods:**

A cross-sectional study based on questionnaire administration was used among women of childbearing age. Data collection was conducted from August to October 2021. A scoring method was applied to evaluate their knowledge level and perceptions about COVID-19 and its impacts on their lives. A binary stepwise logistic regression was performed to determine the sociodemographic determinants of their knowledge level about COVID-19.

**Results:**

A total of 885 women of childbearing age from urban and rural Moramanga areas were interviewed. Approximately 49.8% (441/885) lived in urban areas, and 50.2% (444/885) lived in rural areas. Approximately 35.3% (322/885) of the participants had a good level of knowledge of COVID-19. Multivariate analysis showed that the probability of having a good level of knowledge of COVID-19 had a significant statistical association *(p value < 0.05)* with living in an urban area [AOR: 2.89; 95% CI (1.89–4.42)], telephone ownership [AOR: 1.71; 95% CI (1.16–2.53)], radio ownership [AOR 2.2; 95% CI (1.43–3.38)], watching TV [AOR = 1.95; 95% CI (1.34–2.83)] and reading journal papers [AOR = 3.74 95% CI (1.69–8.27)].

**Conclusions:**

Almost a third of the sampled women of childbearing age had a good level of knowledge of COVID-19. Access to information through telecommunications technologies increases the chances of being better informed about the disease. To avoid the negative repercussions of infectious disease epidemics, it is necessary to improve the awareness of childbearing women about these diseases by taking demographic features of the population into account.

## Background

The severe acute respiratory syndrome coronavirus 2 (SARS-CoV-2), also known as the COVID-19 (coronavirus disease) pandemic, has affected the whole world from December 2019 to the present time. First detected in China, the disease has spread around the world [1–5]. The World Health Organization declared an international public health emergency in January 2020 [1–5]. Due to the “newness” of the disease, in some developed and resource-poor countries alike, in addition to medical measures, strict nonpharmaceutical measures such as containment have been adopted by states and governments to either stop or slow down the circulation of the disease in their territories [6]. Madagascar experienced two epidemic waves of COVID-19 and two states of health emergency from March 2020 to September 2021 [7, 8]. During these health emergencies, in addition to the closure of international borders, internal travel restrictions (between regions), curfews, and partial or total confinements were decreed by the Malagasy government to address the spread of the disease [8, 9].

The COVID-19 epidemic has probably had repercussions on the use of health care and health facilities in Madagascar, as is the case in some parts of the world [10–15]. Such repercussions would have resulted in changes in habits and behavior among the population [16]. In low- and middle-income countries, neonatal and pediatric utilization and attendance declined [13–16] during the pandemic period. Dropouts or interruptions in the use of maternal and child health care (immunization, reproductive health) have been reported in sub-Saharan African countries [15]. Barriers and/or impediments to the uptake of maternal and child health care may represent indirect impacts of the COVID-19 pandemic [10].

Perceptions (of the disease or risk) and knowledge of the population about the disease are barriers that have been identified in several studies as predictors of dropout and/or interruption of care [16]. Knowledge, attitude and Practice (KAP) studies, or studies of people’s knowledge of diseases, are used to identify gaps in people’s knowledge of diseases, and also to improve public support for public health measures such as vaccination [17]. Populations with an acceptable level of knowledge about COVID-19 are more likely to follow preventive measures and protect themselves against the disease [18].

Since 2012, the Institut Pasteur (IPM) of Madagascar has set up a population-based cohort platform, namely, the MHURAM cohort (“*Moramanga* Health survey in Urban and Rural Areas in Madagascar”) in the district of *Moramanga*, located in the middle east area of Madagascar [19]. Within the framework of this cohort, population censuses and follow-ups in 3 municipalities of the Moramanga district, namely, *Moramanga, Ambohibary*, and *Ampasimpotsy*, were conducted. Routine vaccination activities (polio, diphtheria, tetanus, and pertussis measles) in children under 5 years of age or maternal and child health consultations (prenatal consultations, deliveries, postnatal follow-up) and consultations concerning children under 5 years of age may have been disrupted during the epidemic period of COVID-19 in Moramanga. Using information from the MHURAM platform, the aims of the study were to (1) assess the knowledge and perceptions of childbearing women in this region regarding COVID-19 and (2) identify the determinants of their knowledge of COVID-19 levels.

## Methods

### Study design

A cross-sectional, both descriptive and analytical study based on the administration of a population-based questionnaire was conducted between August and October 2021 in two municipalities of the *Moramanga* district.

### Study site

**Fig. 1:**
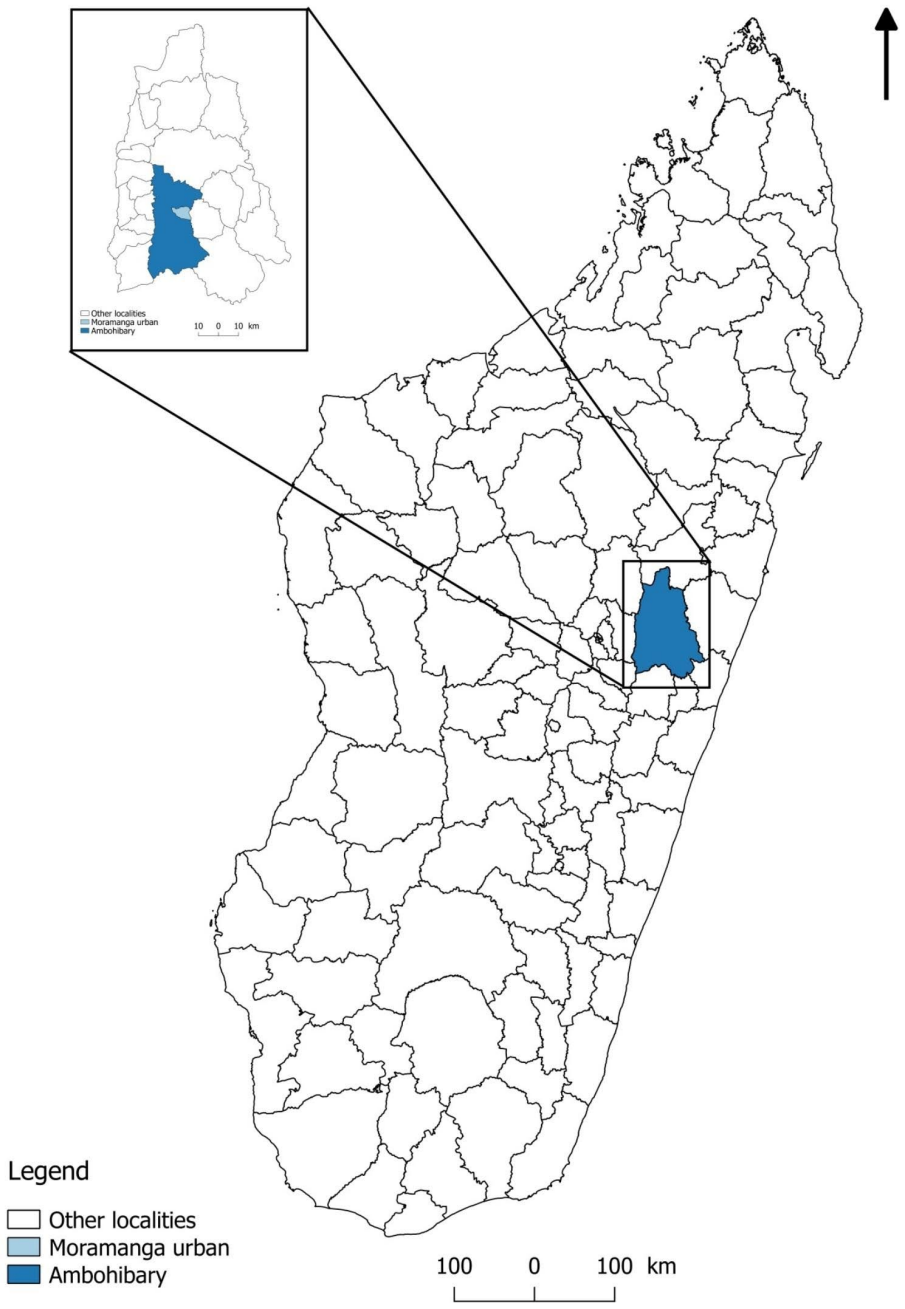
Location of study sites (mapping by Sitraka Rakotosamimanana, SaGEO team, Institut Pasteur de Madagascar, using free Quantum GIS (QGIS) 12.8® software)

The district of Moramanga is part of the *Alaotra Mangoro* region, located in the Middle East area of Madagascar (Fig. 1). The district has 21 municipalities subdivided into 175 *fokontany* (the smallest administrative unit in Madagascar). It has an area of 9336 km2 and is located 112 km east of the capital, Antananarivo. The study sites concerned two municipalities in the district of Moramanga in the Alaotra Mangoro region, namely, the urban municipality of Moramanga, with 12 *fokontany*, and the rural municipality of Ambohibary, with 13 *fokontany*.

### Sample size

Assuming that 50% of the population will correctly answer the questions on perceptions and knowledge about COVID-19, with a precision of 5%, and a cluster design effect estimated at 2 a total of 740 individuals would be required for a 95% confidence interval by using OpenEpi© open source software from MIT, Atlanta, GA, USA [20]. By estimating the proportion of refusals at 15%, the minimal sample size is equivalent to a total of 851 women of childbearing age to be investigated across the two municipalities.

A simple random sampling of individuals to be surveyed was made from the MHURAM database. The addresses of the households to be surveyed were stratified by municipality and then randomly selected. A simple random sampling from a minimum of 426 individuals living in urban municipality of Moramanga and a minimum of 426 individuals living in rural municipality of Ambohibary were realized from the addresses of the households in the database. The team of investigators made home visits to each randomly sampled address. A second random sampling was made if there were several eligible women in the household.

### Participants

Participants consisted of childbearing women aged 12–49 years residing in one of the two study municipalities at the time of data collection. We used the definition of women of childbearing age according to the latest population census in Madagascar, which is between 12 and 49 years [21]. Women with complete addresses in the MHURAM database who agreed to participate were included in the study.

### Ethical considerations

The study received the authorization of the Biomedical Ethics Committee of the Ministry of Health of the Republic of Madagascar No. 060 MSANP/SG/ANM/CERBM on June 29 2021. After reading the information note on the study, the surveyed women signed an informed consent form. For women under the age of 18, the signatories were their legal guardians.

A verbal consent was asked for illiterate women in the presence of a witness this procedure of consent is approved by the Ethics Committee of Ministry of Public Health of the Madagascar Republic.

Survey questionnaire and administration process.

A questionnaire on knowledge and perceptions of COVID-19 was developed in French and then translated into Malagasy and administered in Malagasy to the target population. To obtain spontaneous responses from the respondents, the questions asked about knowledge, attitudes and practices in relation to COVID-19 were open ended.

The questionnaire had three main components, in addition to the sociodemographic characteristics of the participants: (i) Knowledge about COVID-19, (ii) Attitudes and practices during the COVID-19 waves and (iii) Perception of the two epidemic waves of COVID-19.

### Data collection process

#### Data management and analysis

The collected data were first integrated directly by tablet computer into *REDCap®* (Research Electronic Data Capture) [22] and then transferred into Excel®. The database was anonymized. After the coding, transcription and translation of the data, the categorical variables were summarized in frequencies and proportions, and the continuous variables were summarized by their medians with their interquartile range (IQR) or means with standard deviations according to their distribution.

The level of knowledge was determined by assigning scores to the answers provided by the women of childbearing age. A score of 1 was assigned to a correct response, and 0 was assigned to a false or incorrect response. The total scores for each respondent were then summed; after assessing the median of the total scores, the level of knowledge was classified into 2 categories:

- good level: individual scores ≥ the median score.
- low level: individual scores < the median score.

To determine associations between different variables, bivariate (chi-square test, Fisher’s exact test) and multivariate analyses were performed with Stata 13® software. Binomial stepwise logistic regression was used to determine the associations between the behaviors, knowledge scores and perceptions of the study population and their sociodemographic characteristics. The outcome variable was the score level (1 = good/0 = low). Variables with a *p value < = 0.25* were included in the multivariate model and adjusted after a top-down, stepwise process with consideration of the lowest AIC. All the independent variables with a *p value < 0.05* were selected in the final model set.

## Results

### Sociodemographic characteristics of the investigated women

All 885 women of childbearing age approached in the study were included in the study. Nearly 49.8% (441/885) of the participants resided in the urban municipality of Moramanga, and 50.2% (444/885) resided in the rural municipality of Ambohibary. The median age of the respondents was 27 years (IQR = 19–36 years). The mean age of the investigated women in urban areas was 28.8 years (standard deviation SD = 9.8), while in rural areas, it was 27.5 years (SD = 6.5) (Table 1). Regarding educational level, almost 57% (506/885) of the respondents had a secondary education, and approximately 28% (248/885) had attained a primary education. Approximately 12% (106/885) had attained a university level education, and approximately 3% (25/885) had no schooling.

**Table 1:** The demographic characteristics of the investigated women by place of residence

Characteristics	Rural				Urban				Total		
	n		%		n		%		n		%
**Age (years)**											
Mean (Standard deviation)		27.5 (6.5)				28.8 (9.8)				28.1 (10.3)	
**Educational level**											
No education	20		4.5		5		1.1		25		2.8
Primary	182		41		66		15		248		28
Secondary	213		48		293		66.4		506		57.2
Higher	29		6.5		77		17.5		106		12
**Socioprofessional category**											
No professional activity	86		19.4		118		26.8		204		23.1
Primary sector	269		60.6		22		5		291		32.9
Secondary sector	28		6.3		12		2.7		40		4.5
Tertiary sector	61		13.7		289		65.5		350		39.5
**Marital status**											
Married	245		55.2		250		56.7		495		55.9
Widowed	6		1.4		11		2.5		17		1.9
Single	134		30.2		140		31.7		274		31
Divorced/separated	59		13.3		40		9.1		99		11.2

Concerning the socioprofessional activity sector, 23% (204/885) had no professional activity. Almost 39% (350/885) worked in the tertiary sector, 33% (291/885) in the primary sector and 4.5% (40/885) in the secondary sector.

In terms of marital status, 56% (495/885) were married, 31% (274/885) were single, and 11% (99/885) were either divorced or separated from their husbands. Almost 2% (17/885) of the women were widowed.

### Knowledge of COVID-19 and perceptions of the disease and health measures

Of the 885 women surveyed, one woman stated that she had never heard of COVID-19 and was thus categorized in our database as having a low level of knowledge about COVID-19.

### Communication channels for accessing information about COVID-19

Of all the respondents who had heard of COVID-19, nearly 8% (71/884) had heard about it at school. The difference between urban and rural areas was significant (6% in rural areas vs. 10% in urban areas) (*p value = 0.04*). Those who had heard of COVID-19 during awareness campaigns represented approximately 40% (357/884) of the respondents. The difference between rural and urban areas was also statistically significant [29% (130/444) *versus* 51% (227/440) in urban areas *(p value < 0.001)].*

The participant’s entourage is another of the channels through which respondents heard about COVID-19, with 53% (473/884) of respondents. The difference between rural and urban areas was very significant [61% (268/444) in rural areas vs. 39% (172/440) in urban areas] (*p value < 0.001*). Respondents who saw posters about COVID-19 represented 23% (204/884) of the total study population. The proportion of respondents who saw a poster in urban areas was significantly higher than that in rural areas [32% (141/440) vs. 14% (63/444)]. For other communication channels, approximately 90% (789/884) had heard about COVID-19 on the radio (92% of rural respondents vs. 88% of urban respondents).

### Knowledge of COVID-19 symptoms

**Fig. 2:**
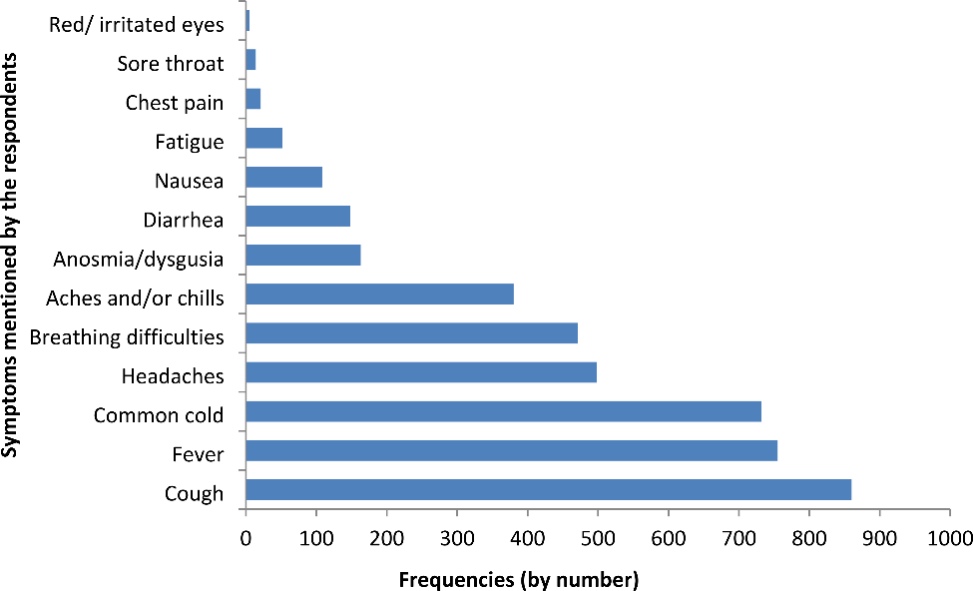
Frequency (number) of symptoms mentioned by the respondents. Legend: x-axis: frequency of respondents’ answers by number; y-axis: symptoms mentioned by respondents

The known symptoms of COVID-19 mentioned spontaneously by the respondents are summarized in Fig. 2. The most frequently mentioned sign of COVID-19 was cough [97% (860/884)], followed by fever [85% (755/884)] and common cold [83% (732/884)]. Headache and breathing difficulties accounted for 56% (498/884) and 53% (471/884)] of respondents’ answers, respectively. Anosmia (loss of smell) and/or dysgeusia (disturbed sense of taste) represented almost 18% (163/884) of the respondents’ answers. Fatigue as a sign of COVID-19 was mentioned by 6% (52/884) of the respondents. Red/irritated eyes as a sign of COVID-19 were also mentioned by rural respondents [0.6% (5/884)].

The modes of transmission of COVID-19 mentioned spontaneously by the interviewees are summarized in Table 2. Direct person-to-person contact was the main mode of transmission of COVID-19 [90% (797/884)], followed by respiratory droplets [89% (786/884)] and aerosols [22% (191/884)]. Soiled/contaminated surfaces were mentioned by 5% (42/884).

**Table 2:** Modes of transmission of COVID-19 mentioned by place of residence

COVID-19 transmission	Rural (n)	%	Urban (n)	%	Total (n)	%
**Direct contact between individuals**						
Yes	409	92 0.1	388	88.2	797	90 0.2
No	35	7.9	52	11.8	87	9.8
**Contaminated surfaces**						
Yes	16	3.6	26	5.9	42	4.8
No	428	96.4	414	94.1	842	95.2
**Aerosols**						
Yes	82	18.5	109	24.8	191	21.6
No	362	81.5	331	75.2	693	78.4
**Respiratory droplets**						
Yes	410	92.3	376	85.5	786	88.9
No	34	7.7	64	14.5	98	11.1

### Type of treatments known by the respondents

In total, 55% (488/884) of the respondents said they had already been infected with COVID-19 (suspected) or knew someone who had. The difference between urban and rural areas was highly significant [48% (213/444) vs. 61% (268/440)] (*p value < 0.001*).

According to 99% of the respondents (878/884), COVID-19 is a disease that can cause death.

A total of 80% (705/884) of the respondents stated that medicines and/or treatments (chemical and traditional) could help cure COVID-19. A proportion of the respondents [17% (147/884)] did not know if there were any specific medicines or treatments to cure the disease.

For the type of treatment known by the respondents for COVID-19, 81% (575/705) mentioned *COVID Organics* or *CVO* [38% (217/575) rural vs. 62% (358/575) urban], which is an herbal treatment developed by the Madagascar Institute of Applied Research based on medicinal plants. Nearly 31% (277/705) mentioned essential oils or medicinal plants [61% (169/277) rural vs. 39% (108/277) urban], and 10% (72/705) mentioned specific chemical treatments [14% (10/72) rural vs. 86% (62/72)]. ED1, an improved traditional treatment based on specific medicinal plants, was mentioned by approximately 5% (34/705) of respondents [9% (3/34) rural vs. 91% (31/34)], while vaccination or vaccines as a treatment of COVID-19 (without specifically mentioning a vaccine name) was mentioned by 3% (20/705) of respondents [95% (31/34) urban].

### Perceptions of COVID-19 waves and disrupts habits during the emergency period

Nearly 99% (873/884) of respondents reported having talked about COVID-19 with their family and friends during the period from March 2020 to April 2021 [100% (444/444) in rural area vs. 97% (428/440) in urban area].

### Disrupted habits during the health emergency/epidemic wave period of COVID-19

Approximately 95% (842/885) of the respondents reported having changed their daily habits during the health emergency period in Madagascar (March 2020-April 2021). Most of them, i.e., 99% (883/884), reported having adopted specific measures in relation to the spread of COVID-19.

### Specific measures against the spread of COVID-19

The specific measures cited by the respondents were inhalation/smoking (with *ravintsara* or other types of plants or essential oils) [60% (533/884)], isolation/limitation of exits [31% (272/884)], frequent hand washing [50% (443/884)] and using masks/mouth covers [62% (547/884)].

### Perception of the severity of epidemic waves

**Fig. 3:**
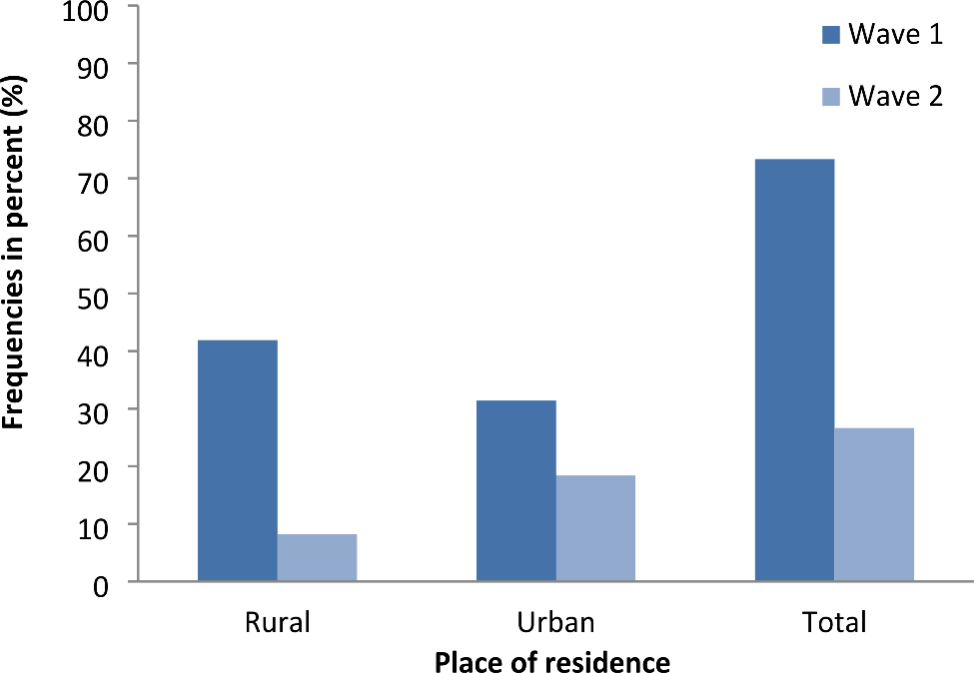
Perception of respondents towards COVID-19 epidemic waves by place of residence. Legend: x-axis: place of residence; y-axis: proportions of respondents’ answers in percentages

The first epidemic wave of COVID-19 ranged from March to October 2020, while the second wave ranged from April to September 2021 (health emergency period). Instead of mentioning precise dates, the respondents spoke of the 2020 wave and the 2021 wave. Regarding the perception of the severity of the two COVID-19 epidemic waves in Madagascar, 73% (649/885) of respondents felt that they had more difficulties during the first wave compared to 27% (236/885) during the second wave (Fig. 3).

By area of residence, nearly 84% (371/444) of respondents living in rural areas felt that the first wave was more serious than the second. In urban areas, this proportion was 63% (278/441). The difference in perception of the severity of the epidemic waves was statistically significant between the two settings (*p value < 0.001*).

### Types of measures hard felt or experienced by the interviewees

The measures most severely experienced or perceived by the respondents were, in order of frequency of response, daytime travel restrictions [90% (796/885)]; confinement [87% (775/885)]; compulsory wearing of masks/mouth covers [24% (216/885)]; curfew [5% (46/885)]; and road closures [3% (28/885)].

### Assessment of the level of knowledge of COVID-19 among women of childbearing age

After scoring, the median score was 11 (IQR; 9–12). For the total of women surveyed (N = 884) who had heard of COVID-19, almost 36% (322/884) had a good level of knowledge with an individual score = > 11) compared to approximately 64% (562/884) who had a low level of knowledge with an individual score < 11.

By area of residence, in urban areas, the median level of knowledge of COVID-19 was 11 (IQR; 9–12). In rural areas, the median was 10 (IQR; 9–12). The association between COVID-19 knowledge scores and residence was statistically significant (*p value < 0.001*) (Fig. 1).

### Determinants associated with the knowledge levels of childbearing women

After an analysis based on a binary stepwise logistic regression [Table 3], the variables significantly associated *(p value* < 0.05) with good or low knowledge of COVID-19 were the place of residence, the possession or not of information access tools and the sector of activity. Indeed, compared to people living in the rural municipality of Ambohibary, those living in the urban municipality had a higher probability of having a good level of knowledge of COVID-19 [adjusted odds ratio (AOR) = 2.89; 95% confidence interval (95% CI) (1.89–4.42)] (*p value < 0.001*). Owning a mobile phone increased the probability of having a good level of knowledge of COVID-19 [AOR = 1.71; 95% CI (1.16–2.53)] (*p value = 0.006*). For the sources of information, owning a radio [AOR = 2.2; 95% CI (1.43–3.38)] (*p value < 0.001*); watching TV [AOR = 1.95; 95% CI (1.34–2.83)] (*p value < 0.001*) and reading journal papers [AOR = 3.74 95% CI (1.69–8.3)] (*p value < 0.001*) increased one’s likelihood of having good level of knowledge of COVID-19. Compared to individuals with no socioprofessional activities, working in the primary sector [AOR = 0.35; 95% CI (0.19–0.64)] (*p value = 0.001*) and the tertiary sector [AOR = 0.48; 95% CI (0.28–0.83)] (*p value = 0.008*) significantly decreased the probability of having a good level of knowledge of COVID-19. Compared to those aged under 21 years, the women aged between 22 and 31, and between 32 and 41 years, had a higher probability of having a good level of knowledge of COVID-19 *(respectively p value 0.014 and 0.02*) (Table 3).

**Table 3:** Determinants of knowledge level towards COVID-19 after multivariable analysis

Variables	Knowledge level				Unadjusted	Adjusted	95% CI		*p value*
					OR	OR			
	good		low				lower	upper	
	**n**	**%**	**n**	**%**					
**Area of residence**									
Rural	80	9.04	364	41.13	1	1			
Urban	242	27.34	199	22.48	5.53	2.89	1.89	4.42	*< 0.001*
**Age category (in years)**									
12 to 21	92	10.4	209	23.62	1	1			
22 to 31	105	11.86	148	16.72	1.61	1.92	1.14	3.25	*0.014*
32 to 41	84	9.49	123	13.9	1.55	2.38	1.37	4.15	*0.002*
42+	41	4.63	83	9.38	1.22	1.35	0.71	2.56	*0.325*
**Marital status**									
Married	178	20.11	317	35.82	1				
Single	105	11.86	169	19.1	1.10				
Widow	8	0.9	9	1.02	1.58				
Separated	31	3.5	68	7.68	0.81				
**Socioprofessional category**									
None	91	10.28	113	12.77	1	1			
Primary	47	5.31	244	25.57	0.23	0.35	0.19	0.64	*0.001*
Secondary	11	1.24	29	3.28	0.47	0.46	0.18	1.14	*0.095*
Tertiary	173	19.55	177	20	1.21	0.48	0.28	0.83	*0.008*
**Ongoing education**									
No	238	26.89	463	52.32	1				
Yes	84	9.49	100	11.3	1.63				
**Educational level**									
None	2	0.23	23	2.6	1				
Primary school	49	5.54	199	22.49	2.83				
College level	230	25.99	276	31.19	9.58				
High level	41	4.63	65	7.34	7.25				
**Mobile phone owning**									
No	55	6.21	218	24.63	1	1			
Yes	267	30.17	345	38.98	3.06	1.71	1.16	2.53	*0.006*
**Access to information**									
**By radio**									
No	53	5.99	112	12.66	1	1			
Yes	269	30.4	451	50.96	1.26	2.2	1.43	3.38	*< 0.001*
**By TV**									
No	123	13.9	406	45.88	1	1			
Yes	199	22.49	157	17.74	4.18	1.95	1.34	2.83	*< 0.001*
**By journal paper**									
No	283	31.98	554	62.6	1	1			
Yes	39	4.41	9	1.02	8.48	3.74	1.69	8.27	*< 0.001*

## Discussion

The aim of our research was to evaluate the knowledge and perceptions of childbearing women in Moramanga regarding COVID-19. In our population, only 36% had a good level of knowledge about the disease. This knowledge was influenced by sociodemographic, and other determinants: living in an urban area, owning a mobile phone, having a radio, watching TV, reading journal papers and being aged between 22 and 41 years old increased the likelihood of having a good level of knowledge of COVID-19 for our study population. On the other hand, working in the primary and tertiary sectors decreased the probability of having a good knowledge level of COVID-19. The present study sheds light on the behavior of a subgroup of a population during an epidemic and can contribute to decision support in the management of care during an epidemic.

The results obtained in studies conducted in East African countries are similar to those of the present study, where the knowledge scores of the general population are low compared to those in West African regions [14, 23], such as in Ethiopia, where almost 43% of the general population investigated in one study had an acceptable knowledge of the disease.As in most African countries, the most common symptoms of COVID-19 reported by respondents were fever, cough, fatigue and cold in both rural and urban areas [24–26]. In southwestern Ethiopia [24], Ivory Coast [25] and Cameroon [26], during outbreaks of COVID-19, fever, dry cough, dyspnoea, myalgia and fatigue were known by the general population as the main clinical symptoms of COVID-19.

Compared to our results, which show that a high proportion of the respondents mentioned direct contact between individuals as a mode of transmission of COVID-19, for the general population in a study in southwestern Ethiopia, respiratory droplets of infected people were the mode of transmission of COVID-19 [26]. In terms of treatments against COVID-19, despite existing chemoprophylactic treatments for COVID-19, traditional, nonpharmaceutical treatments and herbal remedies, *such as COVID organics* or *CVO*, were largely used by respondents during the two waves of the COVID-19 pandemic spanning from 2020 to 2021. The *CVO* cure has been recommended by the Malagasy government as herbal prophylaxis to prevent and treat COVID-19 since April 2020 [27, 28]. This is probably because traditional therapy and herbal remedies are used as popular treatments for many diseases in Madagascar and Africa, both in urban and rural areas [23, 24]. In a context where most households live below the threshold of poverty and where pharmaceutical treatments are particularly expensive for the general population in African countries, traditional treatments are less expensive alternatives or complementary treatments [24, 25, 29, 30]. *COVID-organics*, or *CVO*, has also received much attention from the Malagasy media because the Malagasy government has advocated it as a treatment for COVID-19 since April 2020 [27, 31]. The perception of epidemic waves of COVID-19 is mainly related to the restrictions imposed by the health emergency between 2020 and 2021 among women of childbearing age.

Periods of health emergencies have prompted women of childbearing age in our sample to change their daily habits and adopt specific measures to cope with the spread of COVID-19. The first epidemic wave (March to October 2020) was perceived as more severe than the second (April to September 2021). This perception of the severity of the waves seems to be more related to the impact on women’s daily lives due to restrictive measures taken during the health emergencies than to the fear of the disease. Most people living in Moramanga rural and urban areas work in the informal sector, and restrictive measures such as lockdown and displacement limitations implemented during the day have an impact on the daily income of the population, as in other African countries [23–26, 32, 33].

In terms of other socio-demographic determinants influencing perceptions and knowledge of COVID-19, the area of residence may play a central role, according to our results. Women residing in urban areas have better knowledge of the disease than those residing in rural areas. This is probably because urban women have more access to information about the disease than rural women. This is similar to the results of a study comparing KAP levels on COVID-19 in rural and urban populations in Cameroon [33] and Ethiopia [34], where urban populations have more knowledge about the disease than rural populations. Rural populations have limited access to information channels about COVID-19 [33–36]. In relation to sociodemographic determinants, women working in the primary and tertiary sectors are also more likely to have a lower level of knowledge of the disease than those who are not working. This is probably because women who are working are less aware of COVID-19 due to having a lower level of access to information about COVID-19 than those who are not working.

The other determinants of knowledge of COVID-19 seem to be associated with communication channels (possession of a mobile phone, television, awareness campaigns, etc.) and the knowledge of people who have already documented episodes of COVID-19. These results confirm that people with access to the media and various sources of information have a better knowledge of COVID-19, as seen in studies conducted in Cameroon [33] and Ethiopia [34]. Women aged between 22 and 41 years old may be more concerned about COVID-19 as they tend to have a family; i.e., they would be worried about the health of their young children more so than women in other age groups. Indeed, in 2018, according to the National Institute of Statistics in Madagascar regarding the last population census, the average age at childbirth was 29.4 years nationwide. By place of residence, the average age at childbirth is 28.8 in urban areas *versus* 29.6 in rural areas [21]. This could imply that below these ages, women would be less protective of their surroundings, whereas above the age of 41, the probability of having a young child decreases.

### Limitations of the study

For reasons of accessibility and security, some women living in remote areas of our study were not included in our investigations. Thus, our results were limited by the lower representation of women living in remote areas. The women in remote areas who needed to be investigated were replaced by women living nearby in accessible locations.

## Conclusions

The results of the current study allowed us to draw up a profile of childbearing women with good and poor knowledge of COVID-19 living in the study area. The knowledge and perceptions of COVID-19 among the target population could effectively influence their care-seeking practices during epidemic periods or possible future epidemic waves of COVID-19 or other resurgent diseases. Thus, it is necessary to adapt health messages according to the different subgroups and categories of the population (age category, rural/urban, etc.).

## Data Availability

The datasets generated and/or analyzed during the current study are not publicly available but are available upon request to the corresponding author.

## Abbreviations

COVID-19: Coronavirus disease
MHURAM: Moramanga Health survey in Urban and Rural Areas in Madagascar
REDCap: Research Electronic Data Capture
Sars-COV 2: severe acute respiratory syndrome coronavirus 2
WHO: World Health Organization
